# Supplementary material for: RNA editing enzyme APOBEC3A promotes pro-inflammatory M1 macrophage polarization
Source: Commun Biol. 2021 Jan 22;4:102. doi: 10.1038/s42003-020-01620-x (PMC7822933; doi:10.1038/s42003-020-01620-x)
Supplement: Supplementary file 2 — Supplementary Information [file 42003_2020_1620_MOESM2_ESM.pdf]

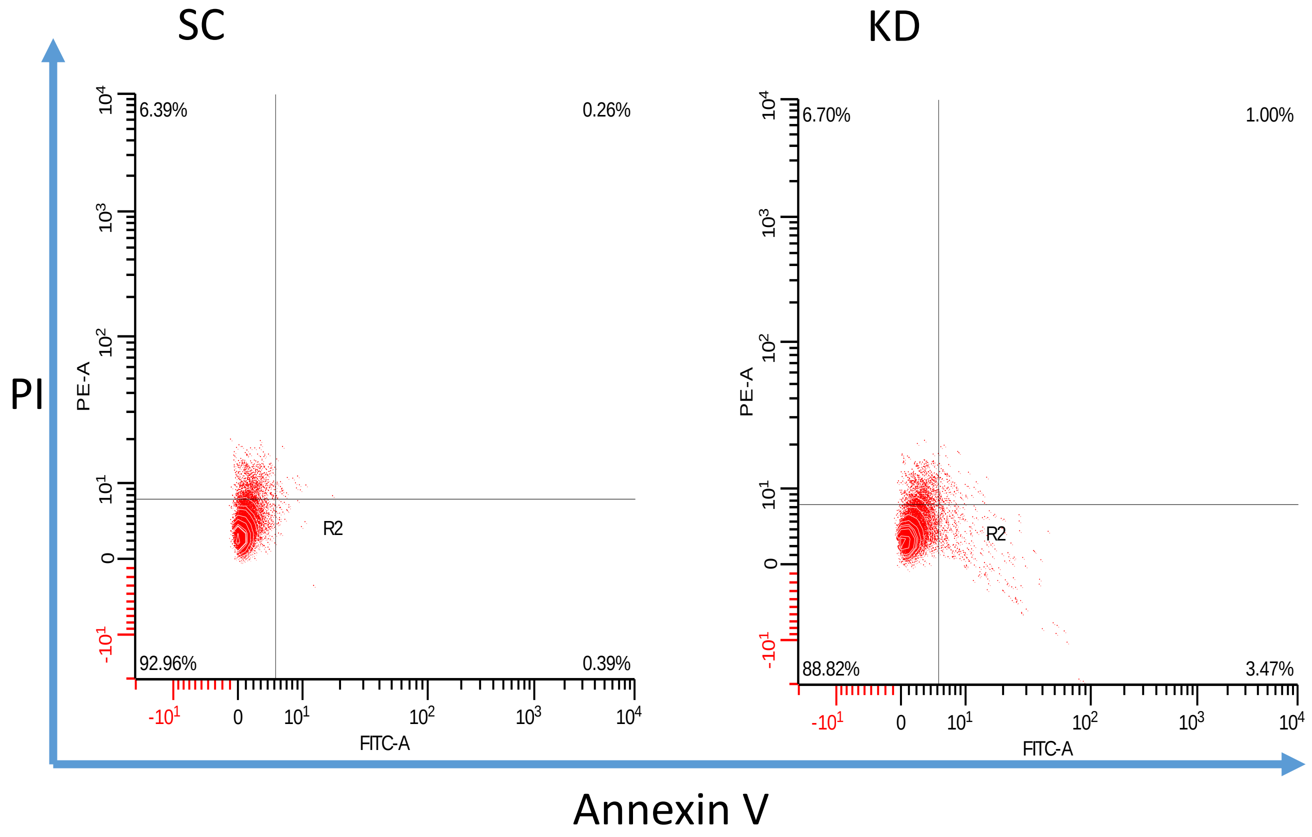

Supp. Figure 1. A3A does not affect cell viability in M1-macrophages. Monocytes isolated from normal donor blood were differentiated to M0 macrophages as described. M0 macrophages, after A3A knock down (or control siRNA transfection, SC), were polarized to M1 macrophages with IFN-ϒ (20ng/ml for 48h) and LPS (50ng/ml for 48h) and evaluated by flow cytometry for cell death using Annexin V and PI. Percent viable cells are similar in SC (93%) and KD (89%).

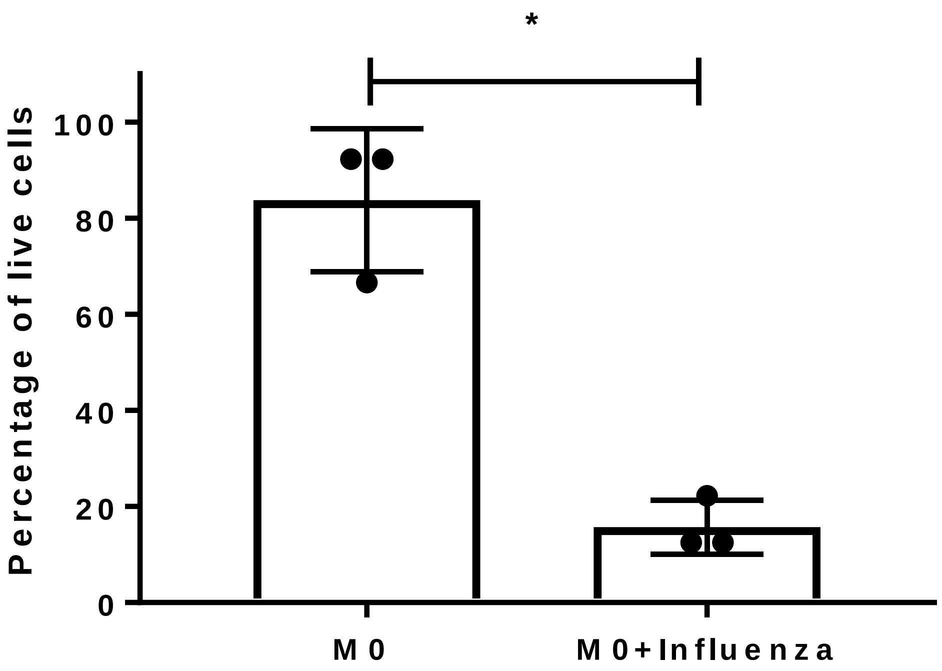

Supp. Figure 2. Influenza virus infection reduces cell viability. (P=0.0119 Paired t-test, two sided; n=3 donors). Cells are harvested 48 hours after influenza or vehicle infection (see Method), and non-viable cells are counted using trypan blue after 50,000-fold dilution

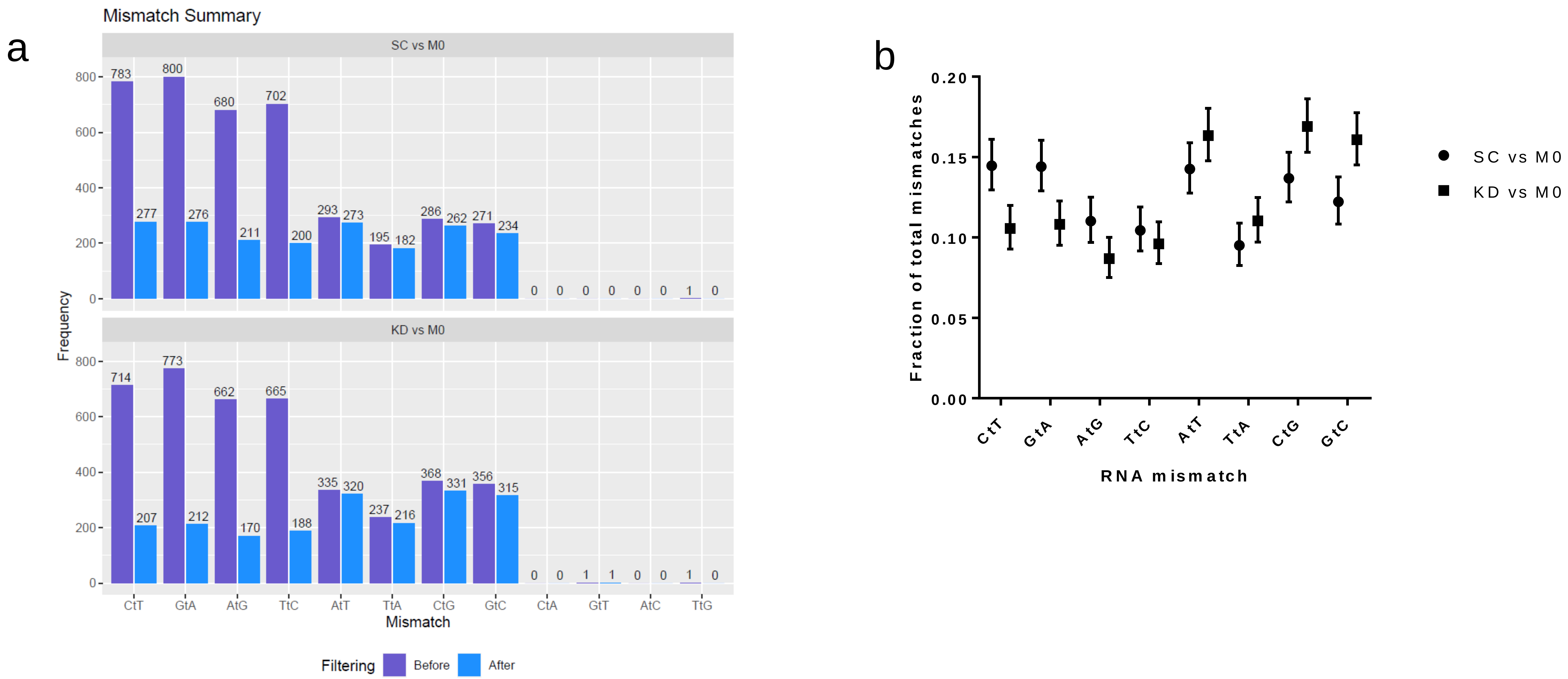

Supp. Figure 3. Global RNA mismatch analysis of RNA Seq data shows the largest fractional decreases in CtT and GtA mismatches, the superset of C>U RNA editing events, upon knockdown of APOBEC3A. a Mismatch frequency before and after germline filtering for the two planned comparisons are shown. The detection process is identical to the one described in the Methods section, except that -1 T/C and stem-loop filters were not employed. Briefly, the first round removes all points with at least a sample having > 0.95 editing level, corresponding to homozygous SNPs. Then SNPs were filtered out if editing levels in all of the samples were below 0.05, higher than 0.95 or values between 0.4 and 0.6 AND are called SNPs according to dbSNP144. Finally, if editing level is less than 5% in any experimental group or increase less than 2-fold, those sites are also excluded. **b** Fractions of each mismatch within each experimental group are shown along with 95% confidence intervals (Wilson/Brown Method, recommended by GraphPad Prism software), excluding CtA, GtT, AtC and TtG that have fractions ~0. The largest decreases were observed in CtT and GtA, the superset of true C>U RNA editing events, when APOBEC3A is knocked down relative to scrambled control M1 macrophages.

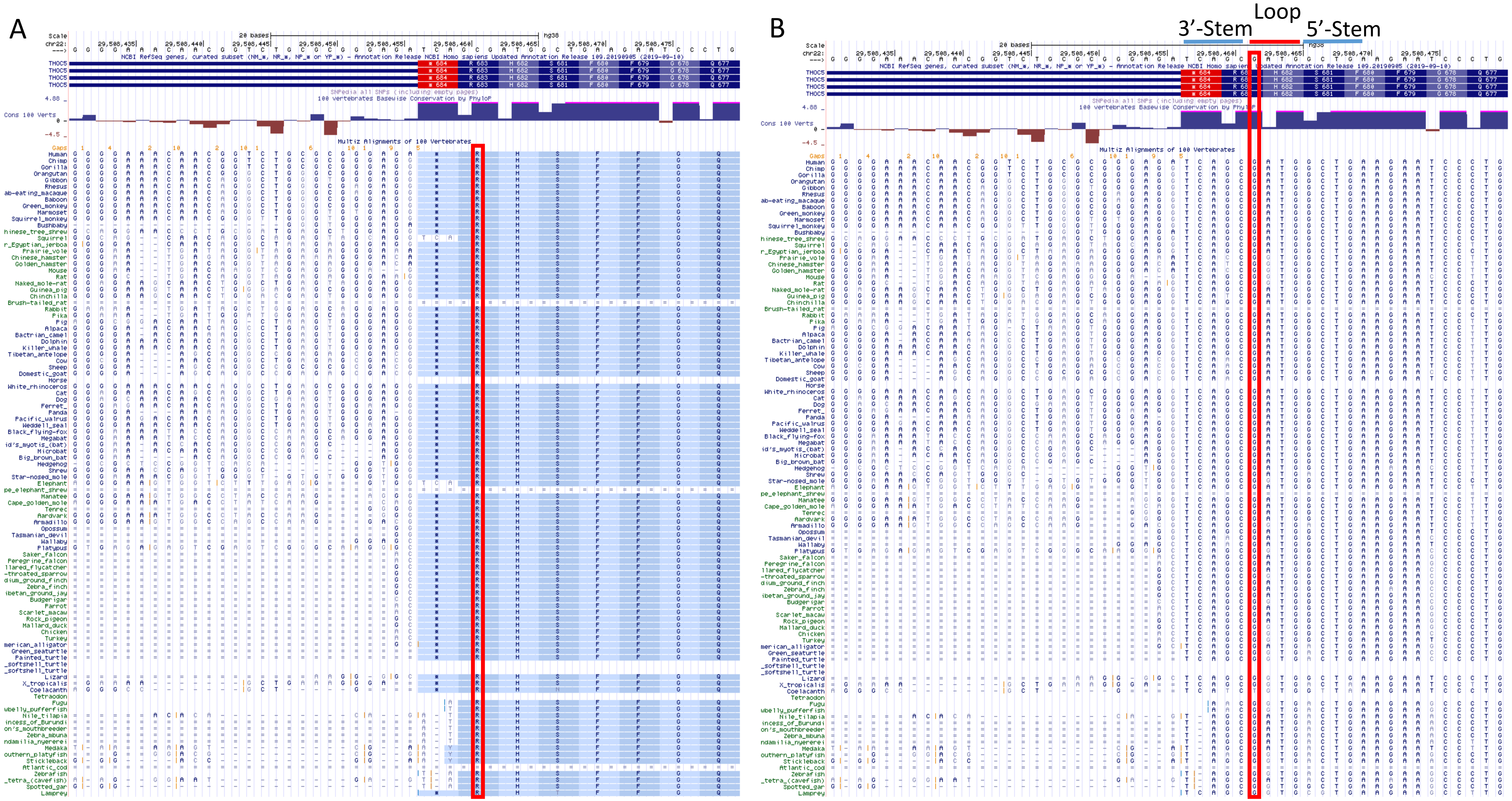

Supp. Figure 4. Conservation of *THOC5* C>U site-specific RNA editing site (c.C2047T:p.R683C) at protein (A) and DNA level (B) is shown. The input for multiple species alignment for producing the conservation state annotations is available at hthttp://hgdownload.soe.ucsc.edu/goldenPath/hg38/multiz100way/alignments/

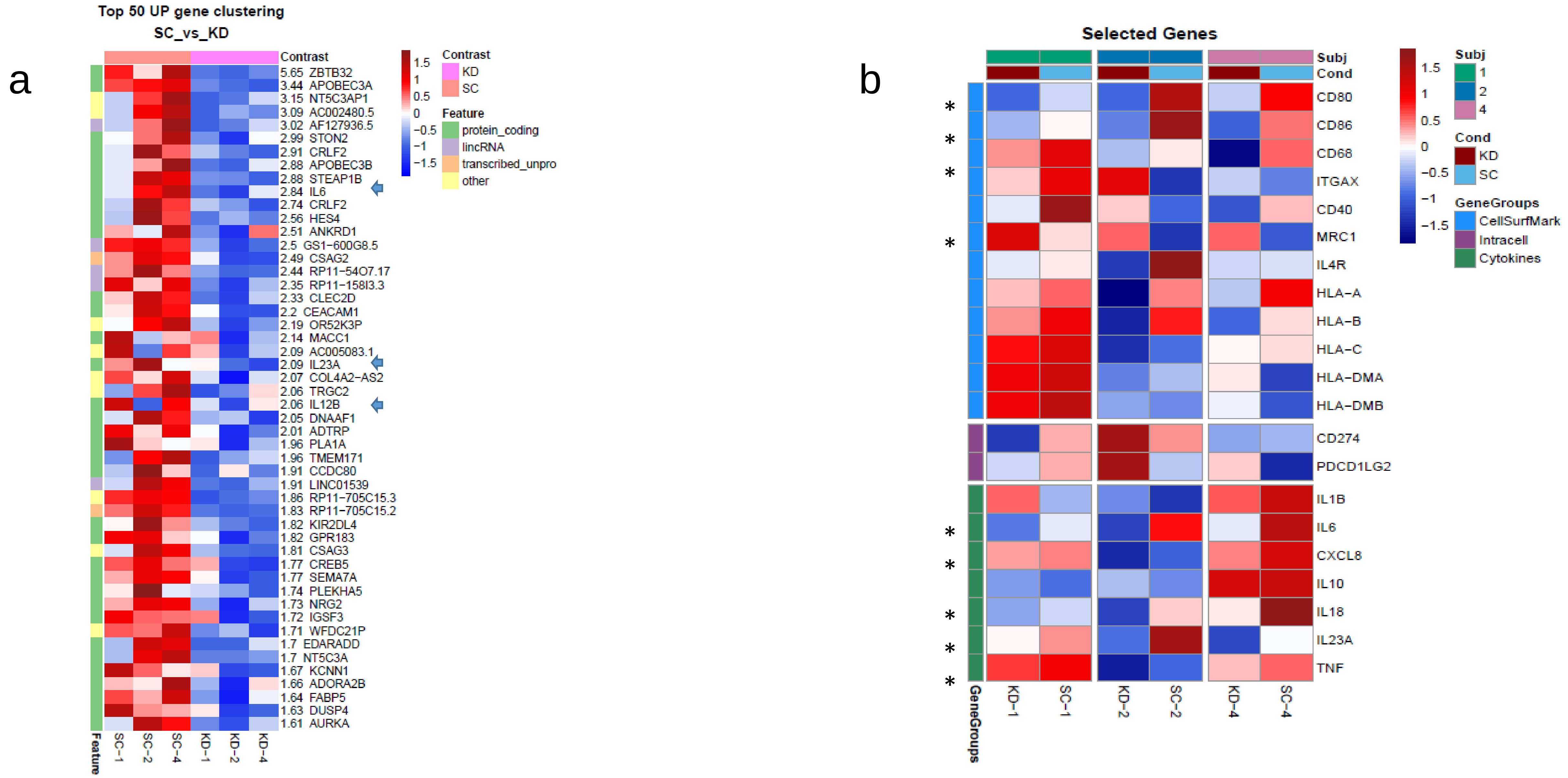

Supp. Figure 5. A3A augments inflammatory gene expression in M1-polarized macrophages. Heatmaps of selected pro-inflammatory genes show greater expression in M1 A3A SC macrophages compared to M1 A3A KD macrophages. **a** Heatmap of the top 50 most upregulated genes in M1 SC samples relative for M1 A3A KD samples include pro-inflammatory genes *IL6, IL23A* and *IL12B* (arrowheads). **b** Expression of *CD80, CD86, CD68, TNF, IL23A, IL18, CXCL8* and *IL6* genes are reduced with A3A KD (* p = <0.05), while MRC1 (CD206), which is associated with M2 polarization, had increased expression with A3A KD.

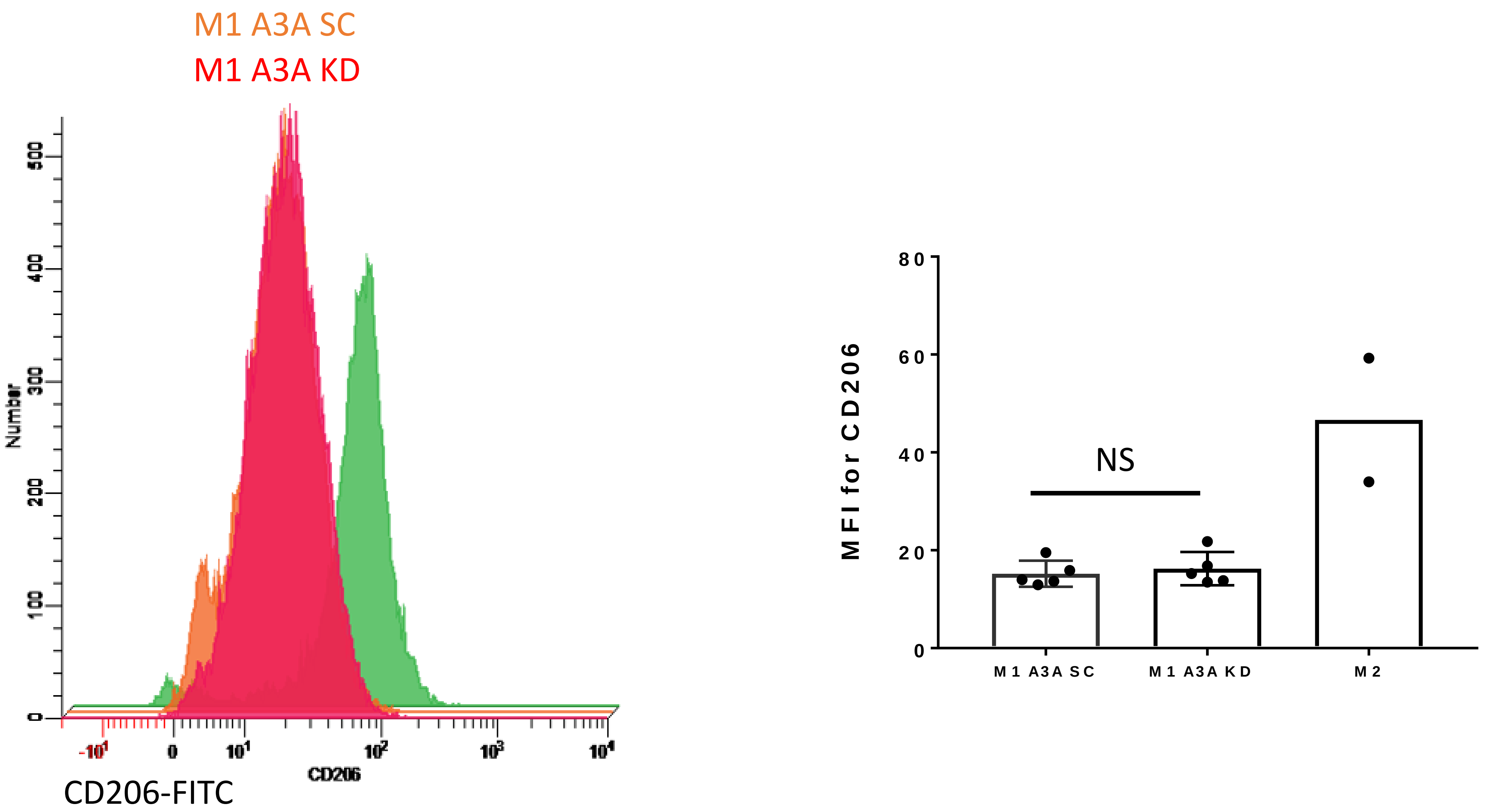

Supp. Figure 6. Knockdown (KD) of A3A does not alter surface expression of CD206, a marker for M2 macrophages The quantification of CD206 (P= 0.3375, Paired t test, two-tailed, n= 5 donors) is made using flow cytometry (see Methods) following M1 polarization in CD33-positive cells. MFI=Mean (geometric) Fluorescent Intensity. M2 macrophage data from a smaller number of donors (n=2) highlight high expression level in M2. Data in scatter dot plots with mean ± SD.
