# Supplementary material for: RNA editing enzyme APOBEC3A promotes pro-inflammatory M1 macrophage polarization
Source: Commun Biol. 2021 Jan 22;4:102. doi: 10.1038/s42003-020-01620-x (PMC7822933; doi:10.1038/s42003-020-01620-x)
Supplement: Supplementary file 3 — Description of Additional Supplementary Files [file 42003_2020_1620_MOESM3_ESM.pdf]

Description of Additional Supplementary Files

File Name: Supplementary Data 1
Description: Annotated list of Macrophage C>U RNA editing events identified in SC vs M0 comparison, and their levels upon KD

File Name: Supplementary Data 2
Description: Macrophage gene expression differences between SC and KD

File Name: Supplementary Data 3
Description: Raw data points used in Figures 1, 2, 6, 7, and Supplementary Figures 2 and 6
