# Supplementary material for: RNA editing enzyme APOBEC3A promotes pro-inflammatory M1 macrophage polarization
Source: Commun Biol. 2021 Jan 22;4:102. doi: 10.1038/s42003-020-01620-x (PMC7822933; doi:10.1038/s42003-020-01620-x)
Supplement: Supplementary file 7 — Reporting Summary [file 42003_2020_1620_MOESM7_ESM.pdf]

nature research

Corresponding author(s): Bora E. Baysal and Brahm Segal

Last updated by author(s): Dec 4, 2020

Reporting Summary

Nature Research wishes to improve the reproducibility of the work that we publish. This form provides structure for consistency and transparency in reporting. For further information on Nature Research policies, see our Editorial Policies and the Editorial Policy Checklist.

Statistics

For all statistical analyses, confirm that the following items are present in the figure legend, table legend, main text, or Methods section.

n/a	Confirmed	
☐	☒	The exact sample size (*n*) for each experimental group/condition, given as a discrete number and unit of measurement
☐	☒	A statement on whether measurements were taken from distinct samples or whether the same sample was measured repeatedly
☐	☒	The statistical test(s) used AND whether they are one- or two-sided<br>*Only common tests should be described solely by name; describe more complex techniques in the Methods section.*
☐	☒	A description of all covariates tested
☐	☒	A description of any assumptions or corrections, such as tests of normality and adjustment for multiple comparisons
☐	☒	A full description of the statistical parameters including central tendency (e.g. means) or other basic estimates (e.g. regression coefficient) AND variation (e.g. standard deviation) or associated estimates of uncertainty (e.g. confidence intervals)
☐	☒	For null hypothesis testing, the test statistic (e.g. *F*, *t*, *r*) with confidence intervals, effect sizes, degrees of freedom and *P* value noted<br>*Give P values as exact values whenever suitable.*
☒	☐	For Bayesian analysis, information on the choice of priors and Markov chain Monte Carlo settings
☒	☐	For hierarchical and complex designs, identification of the appropriate level for tests and full reporting of outcomes
☒	☐	Estimates of effect sizes (e.g. Cohen's *d*, Pearson's *r*), indicating how they were calculated

Our web collection on statistics for biologists contains articles on many of the points above.

Software and code

Policy information about availability of computer code

Data collection	N/A
Data analysis	N/A

For manuscripts utilizing custom algorithms or software that are central to the research but not yet described in published literature, software must be made available to editors and reviewers. We strongly encourage code deposition in a community repository (e.g. GitHub). See the Nature Research guidelines for submitting code & software for further information.

Data

Policy information about availability of data

All manuscripts must include a data availability statement. This statement should provide the following information, where applicable:

- Accession codes, unique identifiers, or web links for publicly available datasets
- A list of figures that have associated raw data
- A description of any restrictions on data availability

Sequence data from RNA_Seq experiments are deposited to GEO repository at https://www.ncbi.nlm.nih.gov/geo/ under accession number GSE146867. Raw data points used for figures 1, 2, 6, 7, and supp. Figures 2 and 6 are provided in a supplemental file.

Field-specific reporting

Please select the one below that is the best fit for your research. If you are not sure, read the appropriate sections before making your selection.

☒ Life sciences ☐ Behavioural & social sciences ☐ Ecological, evolutionary & environmental sciences

For a reference copy of the document with all sections, see nature.com/documents/nr-reporting-summary-flat.pdf

Life sciences study design

All studies must disclose on these points even when the disclosure is negative.

Sample size	No explicit power analysis is used for sample size estimation. We tested whether APOBEC3A has a large effect size on inflammatory marker secretion/expression.
Data exclusions	No data are excluded from reporting of designed experiments.
Replication	Key findings of our paper are replicated by complementary methods including (a) APOBEC3A knockdown efficiency (by qPCR and western blotting), (b) discovery of new RNA editing targets (by high throughput RNA sequencing and Sanger sequencing), (c) suppression of pro-inflammatory phenotype by APOBEC3A knockdown (RNA_Seq, cytokine measurements and flow cytometry).
Randomization	Allocation of anonymous donor cells to experiments was random.
Blinding	No explicit blinding was performed. However, data collection and analysis for RNA_Seq and SeaHorse experiments were performed by different co-authors. Furthermore, our key findings are replicated by complementary methods as explained above.

Reporting for specific materials, systems and methods

We require information from authors about some types of materials, experimental systems and methods used in many studies. Here, indicate whether each material, system or method listed is relevant to your study. If you are not sure if a list item applies to your research, read the appropriate section before selecting a response.

Materials & experimental systems

n/a	Involved in the study	
☐	☒	Antibodies
☒	☐	Eukaryotic cell lines
☒	☐	Palaeontology and archaeology
☒	☐	Animals and other organisms
☒	☐	Human research participants
☒	☐	Clinical data
☒	☐	Dual use research of concern

Methods

n/a	Involved in the study	
☒	☐	ChIP-seq
☐	☒	Flow cytometry
☒	☐	MRI-based neuroimaging

Antibodies

Antibodies used	Western blot: Rabbit polyclonal Anti-PHO1 (APOBEC3A) antibody (Abcam, product number ab262853, 1:1000 dilution), Rabbit polyclonal anti-THOC5 Antibody (Bethyl Laboratories, product number A302-120A, 1:1000 dilution), Mouse monoclonal Anti-SDHB Antibody (Santa Cruz, product number sc-271548, 1:500 dilution) and Mouse monoclonal Anti-beta Actin antibody (Abcam, product number ab49900, 1:1000 dilution).<br>Flow cytometry: The following antibodies were used: Anti-human CD33 PE-Cyanine7 (eBioscience, San Diego, CA), anti-human CD86 APC (BioLegend, San Diego, CA), and anti-human CD206 FITC (BioLegend).
Validation	All antibodies were validated by vendors. Anti-PHO1 (APOBEC3A) antibody is further validated in our experiments based on expected size, high expression in M1 macrophages and decreased expression by siRNA targeting.

Flow Cytometry

Plots

Confirm that:

☒ The axis labels state the marker and fluorochrome used (e.g. CD4-FITC).

☒ The axis scales are clearly visible. Include numbers along axes only for bottom left plot of group (a 'group' is an analysis of identical markers).

☐ All plots are contour plots with outliers or pseudocolor plots.

☒ A numerical value for number of cells or percentage (with statistics) is provided.

Methodology

Sample preparation	Mononuclear cells were recovered from normal donor blood TRIMA leukoreduction filters by density gradient centrifugation. Monocytes were isolated from mononuclear cells using CD14 microbeads and AutoMACS. CD14+ cells were cultured for 1 week with recombinant human macrophage colony stimulating factor to generate M0 macrophages. For M1 or M2 macrophage polarization, M0 cells were treated for two days with recombinant human IFN-γ and LPS or recombinant human IL4, respectively. On day later, M1 cells were transfected with A3A or scramble siRNA. All cells were stained with fluorescence labeled anti-CD33, anti-CD86 and anti-CD206 mAb for 25 minutes in standard flow buffer. After wash, labeled cells were used immediately for data acquisition in flow cytometer.
Instrument	Flow cytometry analysis was conducted on a LSRII flow cytometer.
Software	Winlist 3D version 8.0 (Verity, Topsham ME).
Cell population abundance	At least 20,000 events collected per sample.
Gating strategy	Forward scatter versus side scatter gating was set to include all non-aggregated cells. CD33+ cells from the total cells were gated to obtain CD33+CD86+ or CD33+CD206+ cell populations based on unstained controls.

☐ Tick this box to confirm that a figure exemplifying the gating strategy is provided in the Supplementary Information.
